# 

**Published:** 1907-11-16

**Authors:** 


CONTENTS.

| | PAGE |
|---|---|
| **Medical Men, Medical Papers, and Insurance** | 155 |
| **Some Notes on Progress in Medicine** | 155 |
| **Chronic Diseases of the Joints** | 156 |
| **Annotations—** | |
| Natural Causes of Sudden Death—Diagnosis and Treatment by Correspondence—The Birthday Honours | 157 |
| **Medical Opinion and Movement** | 158 |
| **Hospital Clinics—** | |
| The Senile Cardio-Vascular System. By Professor T. Clifford Allbutt, K.C.B., M.D., F.R.S. | 159 |
| Painless Effusion into the Knee-joint. By Sir William Bennett, K.C.V.O., F.R.C.S. | 161 |
| **Clinical Points—** | |
| Pyrexia in Malignant Disease of the Liver | 165 |
| **Points in Surgery—** | |
| Trigeminal Neuralgia and its Surgical Treatment | 167 |
| **Gynæcology—** | |
| Gynæcology in General Practice | 169 |
| **Diseases of Children—** | |
| Foreign Bodies in the Air Passages | 170 |
| **Laryngology and Rhinology—** | |
| Simulation of Phthisis by Disease of Pharynx and Nose | 171 |
| **Mental Diseases—** | |
| Insanity and Mental Disorder | 172 |
| **Neurology—** | |
| Thalamic Hemiplegia | 173 |
| **Otology—** | |
| Otitis Media in Infancy and Early Childhood | 173 |
| **Ophthalmology—** | |
| The Optic Nerve and the Accessory Sinuses of the Nose | 175 |
| **Points in Pathology—** | |
| On Phagocytosis | 176 |
| **Public Health and Hygiene—** | |
| The Tendency of Certain Diseases to Die out | 177 |
| The Sanitary Organisation of the Territorial Army | 178 |
| **The Royal Army Medical Corps Section—** | |
| The Official Scheme for the Medical Service of the Territorial Army | 179 |
| **Resident Medical Officers' Department—** | |
| Infectious Diseases in General Hospitals | 180 |
| **Tropical Diseases—** | |
| Anæmia in Tropical Diseases | 181 |
| **The Evolution of Medicine—** | |
| The Charité Hospital, Paris | 182 |
| **"The Hospital" Medical Book Supplement, No. 1** | 183 |
| **Hospital Administration—** | |
| Hospital Finance at its Best | 189 |
| Warrington Infirmary | 191 |
| The Registration of Nursing Homes | 192 |
| Medical Students as Nurses—I. | 193 |
| Social and Poor-law Problems | 194 |
| The Graduates' Medical Schools | 194 |

